# Assessment of PDE4 Inhibitor-Induced Hypothermia as a Correlate of Nausea in Mice

**DOI:** 10.3390/biology10121355

**Published:** 2021-12-20

**Authors:** Abigail Boyd, Ileana V. Aragon, Justin Rich, Will McDonough, Marianna Oditt, Daniel Irelan, Edward Fiedler, Lina Abou Saleh, Wito Richter

**Affiliations:** Department of Biochemistry & Molecular Biology and Center for Lung Biology, University of South Alabama College of Medicine, Mobile, AL 36688, USA

**Keywords:** PDE4, cAMP, nausea, emesis, hypothermia, anesthesia

## Abstract

**Simple Summary:**

Type 4 cAMP-phosphodiesterases (PDE4s) comprise a family of four isoenzymes, PDE4A to D, that hydrolyze and inactivate the second messenger cAMP. Non/PAN-selective PDE4 inhibitors, which inhibit all four PDE4 subtypes simultaneously, produce many promising therapeutic benefits, such as anti-inflammatory or cognition- and memory-enhancing effects. However, unwanted side effects, principally, nausea, diarrhea, and emesis, have long hampered their clinical and commercial success. Targeting individual PDE4 subtypes has been proposed for developing drugs with an improved safety profile, but which PDE4 subtype(s) is/are actually responsible for nausea and emesis remains ill-defined. Based on the observation that nausea is often accompanied by hypothermia in humans and other mammals, we used the measurement of core body temperatures of mice as a potential correlate of nausea induced by PDE4 inhibitors in humans. We find that selective inactivation of any of the four PDE4 subtypes did not change the body temperature of mice, suggesting that PAN-PDE4 inhibitor-induced hypothermia (and hence nausea in humans) requires the simultaneous inhibition of multiple PDE4 subtypes. This finding contrasts with prior reports that proposed PDE4D as the subtype mediating these side effects of PDE4 inhibitors and suggests that subtype-selective inhibitors that target any individual PDE4 subtype, including PDE4D, may not cause nausea.

**Abstract:**

Treatment with PAN-PDE4 inhibitors has been shown to produce hypothermia in multiple species. Given the growing body of evidence that links nausea and emesis to disturbances in thermoregulation in mammals, we explored PDE4 inhibitor-induced hypothermia as a novel correlate of nausea in mice. Using knockout mice for each of the four PDE4 subtypes, we show that selective inactivation of individual PDE4 subtypes *per se* does not produce hypothermia, which must instead require the concurrent inactivation of multiple (at least two) PDE4 subtypes. These findings contrast with the role of PDE4s in shortening the duration of α_2_-adrenoceptor-dependent anesthesia, a behavioral surrogate previously used to assess the emetic potential of PDE4 inhibitors, which is exclusively affected by inactivation of PDE4D. These different outcomes are rooted in the distinct molecular mechanisms that drive these two paradigms; acting as a physiologic α_2_-adrenoceptor antagonist produces the effect of PDE4/PDE4D inactivation on the duration of α_2_-adrenoceptor-dependent anesthesia, but does not mediate the effect of PDE4 inhibitors on body temperature in mice. Taken together, our findings suggest that selective inhibition of any individual PDE4 subtype, including inhibition of PDE4D, may be free of nausea and emesis.

## 1. Introduction

Type 4 cyclic nucleotide phosphodiesterases (PDE4s) comprise a group of four isoenzymes, PDE4A to D, that hydrolyze and inactivate the second messenger cAMP. PDE4s are widely expressed throughout mammalian cells and tissues [1,2,3,4,5], and diverse therapeutic benefits result from their non/PAN-selective inhibition. Preclinical studies of PAN-PDE4 inhibitors have established their potent anti-inflammatory, memory- and cognition-enhancing, anti-depressant and anti-psychotic, metabolic, and cardiovascular properties [3,6,7,8,9,10,11,12,13]. However, the clinical application and commercial success of PDE4 inhibitors have been muted due to adverse effects, particularly nausea, diarrhea, and emesis. These adverse effects are characteristic for this class of drugs, leaving only two PDE4 inhibitors currently approved for systemic administration: Roflumilast, for moderate to severe chronic obstructive pulmonary disease, and Apremilast, for the treatment of psoriasis [2]. However, nausea remains the most common side effect of both Roflumilast and Apremilast (frequency of 28.7% and 8.9%, respectively; from [14,15]).

A multitude of studies have now shown that the genetic knockdown or deletion of individual PDE4 subtypes in cells and/or animals produce unique phenotypes, indicating that each PDE4 subtype exerts unique and non-overlapping roles in the body [2,16,17,18,19,20,21,22,23,24,25,26,27]. Thus, the development of subtype-selective PDE4 inhibitors has been proposed to avert the adverse effects of currently available PAN-PDE4 inhibitors [1,28]. While individual PDE4 subtypes have already been identified as therapeutic targets for a variety of inflammatory, cardiovascular, neuronal, or metabolic conditions [2,6,7,9,10,27,29,30,31,32,33,34,35,36], the PDE4 subtype(s) that mediate the adverse effects of PAN-PDE4 inhibitors remain ill-defined. The main obstacle to their identification is that highly subtype-selective PDE4 inhibitors are not yet available to test and address this question in humans. In addition, the tools that are available, such as the genetic deletion or knockdown of PDE4 subtypes, have been restricted to mice and rats, two species that are anatomically unable to vomit, which is the prevailing preclinical correlate of nausea. Further complicating this analysis is the fact that nausea is by far the more prevalent, and hence the more critical adverse effect of PDE4 inhibitor treatment in humans, compared to emesis [14,15]. It is now well-established that nausea is driven by neuronal and molecular pathways that are partly distinct and extend beyond the mechanisms that drive the emetic reflex [37,38,39,40,41]. And, since nausea is, at its core, a feeling or sensation (e.g., stomach awareness), it is difficult to assess in any animal, including in species that are able to vomit. Thus, while dogs or ferrets are obvious choices to study emesis (compared to mice and rats), rodents may yet prove equally useful in assessing nausea, given a proper correlate. 

We have shown recently that PDE4s play a critical role in the autonomic regulation of body temperature in mice and that treatment with PAN-PDE4 inhibitors induces a fast-onset (within 10 min), substantial (up to −5 °C) and long-lasting (up to 5 h) hypothermia in the animals [42]. Intriguingly, an increasing body of research closely correlates nausea with a significant disturbance in thermoregulation in mammals [43,44,45,46,47]. In humans, nausea is frequently associated with signs of hypothermia, including cold sweats, clammy hands, sometimes shivering and may include a modified perception of ambient temperature and cold-seeking behavior (e.g., cold/fresh air), as well as a reduction in core body temperature [37,44]. Impaired thermoregulation parallels nausea induced by a variety of triggers, including motion, poison, surgery/anesthesia (post-operative nausea and vomiting (PONV)), as well as drug/chemotherapy- or radiation-treatment in humans. Despite the variety of triggers for nausea, they are all consistently associated with hypothermia in various animal species, whether they possess the emetic reflex (e.g., ferrets and shrews) or not (e.g., mice and rats), and can be similarly detected by a reduction in the animals’ core body temperature, cutaneous vasodilation (e.g., tail skin vasodilation), reduced thermogenesis and/or cold-seeking behaviors [43,44,45,47,48,49]. Thus, diverse mammalian species share neuronal mechanisms that link nausea to hypothermia, thus providing a valuable correlate to study nausea. The relationship between nausea and hypothermia appears reciprocal, in that nausea, such as upon induction of motion sickness, predisposes to hypothermia [50,51], and *vice versa*, forced (externally-induced) hypothermia can trigger or predispose to nausea [46]. 

Utilizing the close association between nausea and hypothermia, the present study was designed to assess core body temperature in mice as a potential correlate of nausea and emesis induced by PDE4 inhibitors in humans. We aimed to elucidate the role of individual PDE4 subtypes in mediating these adverse effects and to begin exploring their molecular mechanism(s). Finally, we contrast our findings on PDE4 inhibitor-induced hypothermia with the effects of similar treatments and PDE4 subtype ablation on the duration of Ketamine/Xylazine-induced anesthesia [52], a paradigm previously proposed as a correlate of the adverse effects of PDE4 inhibitors in animals. 

## 2. Materials and Methods

### 2.1. Drugs

RS25344 [53] (1-(3-nitrophenyl)-3-(pyridin-4-ylmethyl)pyrido[2,3-d]pyrimidine-2,4-dione) was obtained from Santa Cruz Biotech (Santa Cruz, CA, USA), Ondansetron ((RS)-1,2,3,9-Tetrahydro-9-methyl-3-(2-methylimidazol-1-ylmethyl)carbazol-4-one) from Sigma-Aldrich (St. Louis, MO, USA) and YM976 [54] (4-(3-chlorophenyl)-1,7-diethylpyrido[2,3-d]pyrimidin-2-one) from Tocris/Bio-Techne (Minneapolis, MN, USA). Rolipram [55] (4-(3-cyclopentyloxy-4-methoxyphenyl)pyrrolidin-2-one), Piclamilast [56,57] (RP73401; 3-(Cyclopentyloxy)-N-(3,5-dichloropyridin-4-yl)-4-methoxybenzamide), Roflumilast [58,59,60] (3-(cyclopropylmethoxy)-N-(3,5-dichloropyridin-4-yl)-4-(difluoromethoxy)benzamide), Clonidine (N-(2,6-dichlorophenyl)-4,5-dihydro-1H-imidazol-2-amine), Yohimbine (methyl (1S,15R,18S,19R,20S)-18-hydroxy-1,3,11,12,14,15,16,17,18,19,20,21-dodecahydroyohimban-19-carboxylate) and Metoclopramide (4-amino-5-chloro-N-[2-(diethylamino)ethyl]-2-methoxybenzamide) were from Cayman Chemical (Ann Arbor, MI, USA). Metoclopramide was dissolved in 1% methylcellulose in water and administered via oral gavage. Clonidine, Yohimbine, Ondansetron and all PDE inhibitors were applied by intraperitoneal (i.p.) injection (100 µL per 20 g body weight). The drugs were initially dissolved in DMSO, and subsequently diluted into phosphate-buffered saline (PBS), pH 7.4, containing final concentrations of 5% DMSO and 5% Cremophor EL (Millipore Sigma, St. Louis, MO, USA). 

### 2.2. Animals

All mice were maintained on a C57BL/6 background and in a temperature-controlled (22–23 °C) vivarium with a 12 h light/dark cycle. Animals were group-housed up to four mice per cage and had ad libitum access to food and water. Adult mice ≥18 g of body weight and ≥10 weeks of age were used for experimentation by evenly and randomly dividing cage littermates into experimental groups. Wildtype C57BL/6 mice were generated in-house using breeders obtained from Charles River Laboratories (Wilmington, MA, USA). Mice carrying genetic deletions of PDE4A [21], PDE4B [61] or PDE4D [62] were generated by Drs. S.-L. Catherine Jin and Marco Conti (Stanford University, CA; [16]), whereas PDE4C knockout mice (Pde4c^tm1.1(KOMP)Wtsi/J^) were generated by the National Institutes of Health (NIH) Knockout Mouse Program (KOMP). All PDE4 knock-out mouse lines were distributed via the Mutant Mouse Resource and Research Centers (MMRRC, http://www.mmrrc.org) (accessed on 18 December 2021) of the University of California at Davis. Experimenters were blinded to the genotypes of the mice and the identity of injected drugs until data acquisition and analyses were completed. All experiments and procedures were approved by the University of South Alabama Institutional Animal Care and Use Committee and were conducted in accordance with the guidelines described in the Guide for the Care and Use of Laboratory Animals (National Institutes of Health, Bethesda, MD, USA). For euthanasia, EUTHASOL^®^ Euthanasia Solution (Patterson Veterinary, Greeley, CO, USA) was injected i.p., followed by cervical dislocation.

### 2.3. Duration of Ketamine/Xylazine Anesthesia

**Figure 1:**
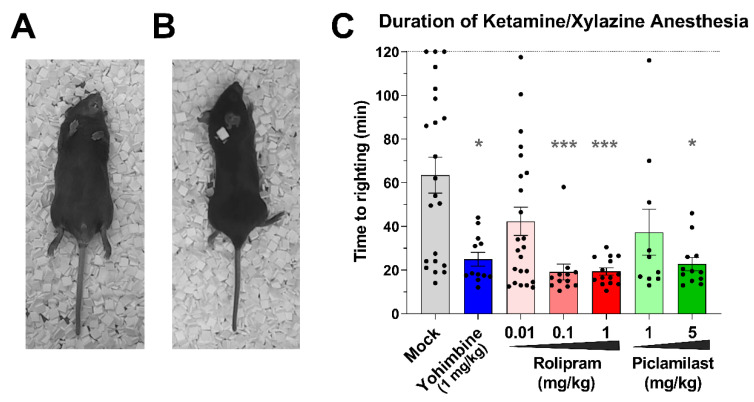
**PAN-selective PDE4 inhibition shortens the duration of Ketamine/Xylazine-induced anesthesia via α_2_-adrenoceptor antagonism.** (**A**,**B**) Representative images illustrating the measurement of “Time to righting”. Upon induction of anesthesia and the resulting loss of righting (~3 min after Ketamine/Xylazine injection), the unconscious mice are placed on their backs (**A**). As the anesthetic effect of Ketamine/Xylazine wears off and the animals awaken, the righting reflex, an automatic reaction to move the body to its normal/prone position, kicks in and the mice turn onto their abdomen (**B**). The time from loss of righting to the time of first righting is recorded. (**C**) Mice were anesthetized with a combination of Ketamine (80 mg/kg) and Xylazine (10 mg/kg) administered by intraperitoneal injection. Ten minutes later, the animals were injected intraperitoneally with the α_2_-adrenoceptor antagonist Yohimbine (1 mg/kg), the PAN-PDE4 inhibitors Rolipram (0.01, 0.1, or 1 mg/kg) or Piclamilast/RP73401 (1 or 5 mg/kg) or solvent control (Mock). The mice were then placed in dorsal recumbency and the time to first righting was measured. Data represent the mean ± SEM. Statistical analysis was determined using the Kruskal–Wallis test followed by Dunn’s post hoc test and is indicated as * (*p* < 0.05) or *** (*p* < 0.001).

The duration of Ketamine/Xylazine-induced anesthesia was measured as described previously [52] with minor modifications. In short, mice were anesthetized with a combination of Ketamine (80 mg/kg) and Xylazine (10 mg/kg) administered by intraperitoneal (i.p.) injection. Upon loss of righting (2–3 min), the mice were then placed in dorsal recumbency and the time to first righting was measured (see Figure 1A,B). Experiments were ended at 120 min after Ketamine/Xylazine injection, and sleep duration for animals that did not right themselves by that time was recorded as 120 min. To probe the effect of drug treatment, the α_2_-adrenoceptor antagonist Yohimbine, the PAN-PDE4 inhibitors Rolipram or Piclamilast/RP73401 or Mock/solvent control were injected (i.p.) into anesthetized mice at 10 min after Ketamine/Xylazine administration. 

### 2.4. Measurement of Core body Temperature

Core body temperature was measured as described [42] using a thermocouple thermometer (MicroTherma 2T) with mouse rectal probe (RET-3), both from Braintree Scientific (Braintree, MA, USA), following the manufacturer’s instructions. 

### 2.5. Data and Statistical Analysis

Statistical analyses were performed using the GraphPad Prism 9 software (GraphPad Software Inc, San Diego, CA, USA). To compare two treatment groups, the Mann–Whitney test with 95% confidence interval was applied. To determine differences between more than two treatment groups, the Kruskal–Wallis test followed by Dunn’s post hoc test was used. For time-course experiments, two-way ANOVA with Sidak’s post hoc test was applied. Statistical differences are shown as # (not significant; *p* > 0.05), * (*p* < 0.05), ** (*p* < 0.01) and *** (*p* < 0.001). All data are expressed as the mean ± SEM and n numbers indicate the number of individual animals assessed and are represented by individual dots in the scatter plots.

## 3. Results

### 3.1. Selective Ablation of PDE4D, but Not Ablation of PDE4A, PDE4B, or PDE4C Mimics the Effect of PAN-PDE4 Inhibitors to Shorten the Duration of Ketamine/Xylazine-Induced Anesthesia in Mice

Xylazine is thought to promote anesthesia *via* its agonism of α_2_-adrenoceptors present at presynaptic sites. Activation of these G_i_-coupled receptors leads to inhibition of adenylyl cyclase activity and a decrease of intracellular cAMP levels, which in turn impairs the release of various neurotransmitters and the resulting suppression of neuronal signaling facilitates anesthesia [63]. As shown previously, treatment with PAN-PDE4 inhibitors counteracts the hypnotic effects of Xylazine, reflected in a shorter duration of Ketamine/Xylazine-induced anesthesia, in various animal species [52,63,64,65,66,67], given that PDE4 inhibition increases cAMP signaling and thus acts as a physiologic antagonist of α_2_-adrenoceptor signaling. As shown in Figure 1C, the archetypal PDE4 inhibitor Rolipram, as well as the second-generation PDE4 inhibitor Piclamilast/RP73401, both shorten the duration of Ketamine/Xylazine anesthesia in mice, although with distinct potencies. As little as 0.1 mg/kg Rolipram significantly shortens Ketamine/Xylazine-induced anesthesia, whereas doses higher than 1 mg/kg Piclamilast are required to produce a significant effect.

**Figure 2:**
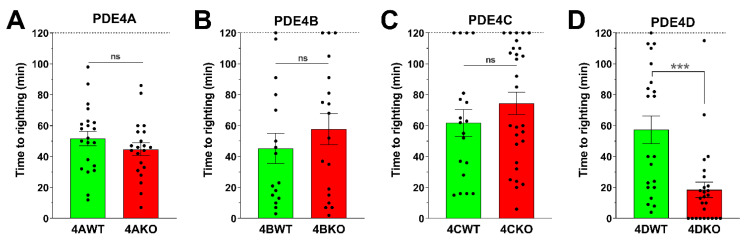
**Genetic ablation of PDE4D, but not ablation of PDE4A, PDE4B or PDE4C, shortens the duration of Ketamine/Xylazine-induced anesthesia in mice.** Mice deficient in PDE4A (**A**), PDE4B (**B**), PDE4C (**C**), or PDE4D (**D**), and their respective wildtype littermates, were anesthetized with a combination of Ketamine (80 mg/kg) and Xylazine (10 mg/kg) administered by intraperitoneal injection. Upon loss of righting (~3 min), the mice were placed in dorsal recumbency and the time to first righting was measured. Mice that never lost the righting reflex were counted as 0 min. Data represent the mean ± SEM. Statistical significance was determined using the Mann–Whitney test with 95% confidence interval and is indicated as *** (*p* < 0.001) or ns (not significant; *p* > 0.05).

Shortening the duration of Ketamine/Xylazine-induced anesthesia has previously been suggested as a physiological correlate of the emetic potential of PDE4 inhibitors, given that both PAN-PDE4 inhibitors as well as α_2_-adrenoceptor antagonists, such as Yohimbine, induce emesis in ferrets and that treatment of ferrets with α_2_-adrenoceptor agonists, such as Clonidine, alleviates PDE4 inhibitor-induced vomiting [64,65]. The duration of Ketamine/Xylazine-induced anesthesia has subsequently been used as a correlate to assess the emetic potential of PDE4 inhibitors in species that are unable to vomit, including mice and rats [52,63]. In a landmark study probing the critical question of which PDE4 subtypes may be involved in the emetic effects of PDE4 inhibitors, it was shown that in mice the genetic ablation of PDE4D, but not the ablation of PDE4B, shortens the duration of Ketamine/Xylazine anesthesia, mimicking the effect of PDE4 inhibitors [52]. This finding has led to the conclusion that PDE4D inactivation mediates the emetic effects of PDE4 inhibitors and is irrevocably tied to inducing emesis. This, in turn, has led to efforts to develop PDE4 inhibitors with selectivity for PDE4B over PDE4D [32,68,69,70,71,72,73], at the same time forgoing numerous therapeutic benefits that may be derived from PDE4D inhibition. Here, we confirm and extend the finding that PDE4D is the sole and principal PDE4 subtype involved in shortening the duration of Ketamine/Xylazine-induced anesthesia in mice. As shown in Figure 2, selective ablation of either PDE4A, PDE4B, or PDE4C in mice has no effect on the duration of Ketamine/Xylazine-induced anesthesia (Figure 2A–C), whereas selective ablation of PDE4D (Figure 2D) shortens anesthesia to levels similar to the administration of PAN-selective PDE4 inhibitors (see Figure 1C). While recent studies have confirmed the value of the Ketamine/Xylazine anesthesia test as a model of α_2_-adrenoceptor antagonism, its value as a physiological correlate of nausea and/or emesis in humans is less clear [67]. We thus explored alternative correlates of emetic potential in mice.

### 3.2. Treatment with PAN-PDE4 Inhibitors Induces Hypothermia in Mice

**Figure 3:**
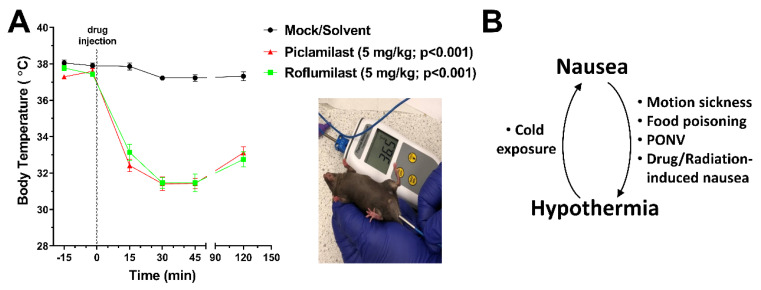
**Treatment with PAN-PDE4 inhibitors induces hypothermia.** (**A**) The core body temperature of mice was measured at the indicated time points prior to and after injection of the PAN-PDE4 inhibitors Piclamilast/RP73401 and Roflumilast (both 5 mg/kg; i.p.) or solvent control. Data represent the mean ± SEM. Statistical significance was determined using two-way ANOVA with Sidak’s post hoc test. The picture inset illustrates the measurement of core body temperature using a rectal thermometer. (**B**) Scheme illustrating the reciprocal relationship between nausea and hypothermia frequently observed in humans and animals. Nausea induced by motion, poison, surgery/anesthesia (post-operative nausea and vomiting (PONV)), drug- or radiation treatment is frequently accompanied by hypothermia. Conversely, aberrant body temperature regulation (including cold exposure) can predispose to nausea [44,45,47,48].

Prior studies have shown that treatment with PAN-PDE4 inhibitors induces a fast-onset, substantial, and long-lasting hypothermia in various animal species, including mice, rats, and rabbits [42,74,75]. As shown previously [42], this is a class effect of PDE4 inhibitors as it is induced by various, structurally distinct PAN-PDE4 inhibitors (see Figure 3A). Intriguingly, an increasing body of evidence causally links nausea and emesis with hypothermia [44,45,47]. Hence, we explored the measurement of core body temperatures to re-evaluate the association of individual PDE4 subtypes with emetic potential. 

### 3.3. PDE4 Inhibitor-Induced Hypothermia Results from the Concurrent Inhibition of Multiple PDE4 Subtypes

**Figure 4:**
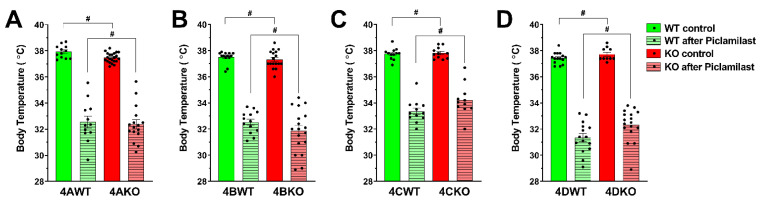
**PDE4 inhibitor-induced hypothermia results from the concurrent inactivation of multiple (at least two) PDE4 subtypes**. The core body temperature of PDE4 knockout (KO, red bars) mice and their respective wildtype (WT, green bars) littermates was measured just prior to (control, solid colors) and again 30 min after i.p. injection of the PAN-PDE4 inhibitor Piclamilast/RP73401 (after Piclamilast; striated bars; 5 mg/kg). Injection of Piclamilast produced a statistically significant drop in body temperature in all genotypes (*p* < 0.01 for 4CKO; *p* < 0.001 for all others). Conversely, the body temperature of mice deficient in (**A**) PDE4A (4AKO), (**B**) PDE4B (4BKO), (**C**) PDE4C (4CKO) or (**D**) PDE4D (4DKO) was not different from that of their respective wildtype (WT) littermates either prior to or after injection of the PDE4 inhibitor Piclamilast. Data represent the mean ± SEM. Statistical significance was determined using the Mann–Whitney test with 95% confidence interval and is indicated as # (*p* > 0.05).

To determine if one of the four PDE4 subtypes (PDE4A to D) is predominantly responsible for the PDE4 inhibitor-induced hypothermia phenotype, we compared the body temperature of mice deficient in PDE4A, PDE4B, PDE4C, or PDE4D to the body temperature of their respective wildtype littermates. As shown in the solid columns of Figure 4A–D, genetic ablation of any of the four PDE4 subtypes did not significantly change the baseline body temperature of mice. To exclude the possibility that compensatory changes may have obscured the loss of one PDE4 in the control of body temperature, the effect of treatment with the PDE4 inhibitor Piclamilast (5 mg/kg, i.p.) was then tested in each knockout strain and their respective wildtype littermate controls. As shown in Figure 4A–D, treatment with a high dose of Piclamilast produced substantial hypothermia in each knockout strain that was similar in extent to that of the matching wildtype controls. Together, these data suggest that ablation of any individual PDE4 subtype does not cause hypothermia, which instead likely results from the simultaneous inhibition of multiple (at least two) PDE4 subtypes. How many and which PDE4 subtypes are involved in mediating the effect of PAN-PDE4 inhibitors on body temperature regulation, and thus associate with emetic potential, remains to be determined.

**Figure 5:**
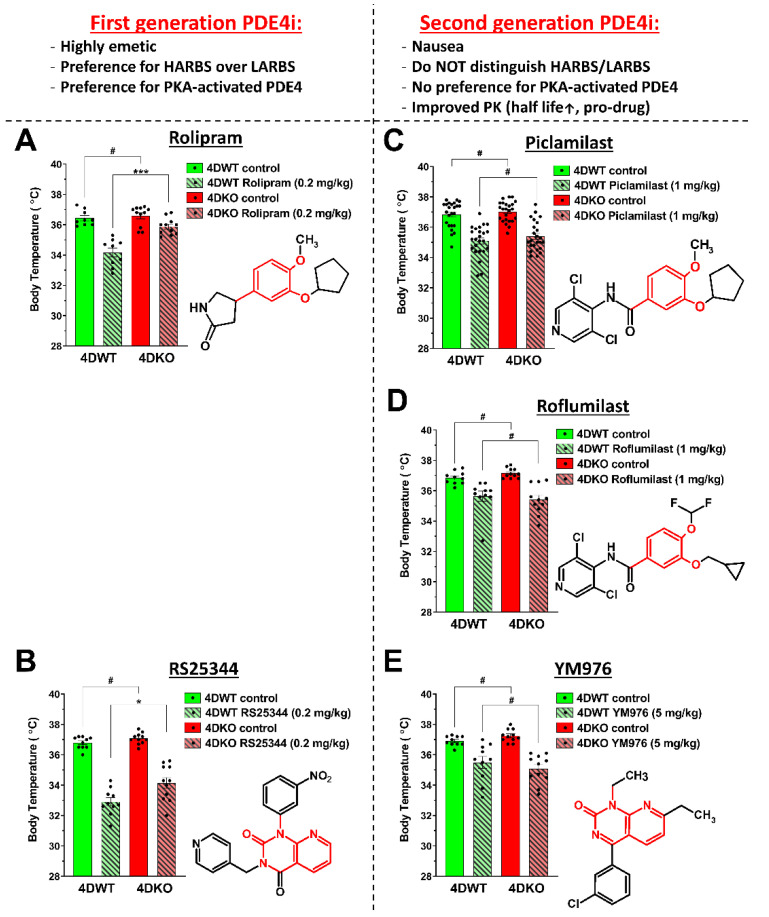
**Genetic deletion of PDE4D in mice alleviates the hypothermia induced by first-generation PDE4 inhibitors Rolipram and RS25344**. (**A**–**E**) Core body temperatures of PDE4D knockout (4DKO) mice and their respective wildtype (4DWT) littermates were measured just prior to (control) and again 30 min after i.p. administration of the PAN-PDE4 inhibitors (**A**) Rolipram (0.2 mg/kg), (**B**) RS25344 (0.2 mg/kg), (**C**) Piclamilast/RP73401 (1 mg/kg), (**D**) Roflumilast (1 mg/kg) or (**E**) YM976 (5 mg/kg). Data represent the mean ± SEM. Statistical significance was determined using the Mann–Whitney test with 95% confidence interval and is indicated as # (p > 0.05; not significant), * (*p* < 0.05) or *** (*p* < 0.001). The chemical structures of the PAN-PDE4 inhibitors tested are shown for comparison. Compounds are grouped into the first-generation PDE4 inhibitors, Rolipram and RS25344 (**A**,**B**), on the left, and the second-generation PDE4 inhibitors, Piclamilast, Roflumilast and YM976 in (**C**–**E**), on the right. First-generation inhibitors are distinguished from second-generation drugs by high emetic potential, selectivity for HARBS (High-affinity Rolipram-binding state—a unique conformation of PDE4 proteins) and their preference for inhibition of PKA-phosphorylated/activated PDE4 [12,53,59,76]. While all listed PDE4 inhibitors do engage in paradigms that require brain-penetrance (e.g., anesthesia, hypokinesia, hypothermia [42,77]), the effects of Rolipram and RS25344 appear more rapid in onset and more potent compared to second-generation PAN-PDE4 inhibitors, suggesting that Rolipram and RS25344 exhibit a more rapid and profound brain-penetrance.

As far as the role of individual PDE4 subtypes, and in particular PDE4D, is concerned, the two correlates of emetic potential studied here, hypothermia (Figure 4) and the duration of α_2_-adrenoceptor-dependent anesthesia (Figure 2), produce contrary results. α_2_-adrenoceptor-dependent anesthesia is exclusively mediated by inactivation of PDE4D, whereas hypothermia is not. Given the lack of any effect of PDE4D ablation on hypothermia, we wished to exclude the possibility that the substantial hypothermia induced by a high dose of Piclamilast (5 mg/kg; Figure 4) may have overshadowed a potential, more subtle effect of genetic PDE4D ablation on body temperature regulation. Thus, we initially repeated body temperature measurements in PDE4D-KO mice and their respective wildtype controls using submaximal doses of the PAN-PDE4 inhibitors Piclamilast and Rolipram as follow-up experiments. Intriguingly, the dose of the PDE4 inhibitors used and/or the amplitude of hypothermia induced by PDE4 inhibitor treatment did not alter the pattern of responses in PDE4D-WT *versus* PDE4D-KO mice. For example, upon treatment with Piclamilast, there was no difference in the levels of hypothermia between PDE4D-WT and PDE4D-KO mice whether a high dose of the drug was used and hypothermia was more substantial (5 mg/kg Piclamilast; −5°C; Figure 4D) or if a lower drug dose was used, producing a more subtle hypothermia (1 mg/kg Piclamilast; −2 °C; Figure 5C). However, we observed that the type of PDE4 inhibitor used affected responses in PDE4D-WT and PDE4D-KO mice. Upon treatment with Piclamilast, Roflumilast, or YM976 (Figure 5C–E), the level of hypothermia induced by the inhibitors was the same in PDE4D-WT and PDE4D-KO mice. Conversely, PDE4D-KO mice were partially protected from hypothermia induced by treatment with Rolipram or RS25344 (Figure 5A,B).

The separation of PDE4 inhibitors into one group that produces distinct effects in PDE4D-WT/KO mice (Rolipram and RS25344) and another group that does not (Piclamilast, Roflumilast, YM976) is not dependent upon the chemical core structure of the drugs (highlighted in red in Figure 5A–E), given that Roflumilast and Piclamilast are derivatives of Rolipram and that YM976 is a derivative of RS25344 (Figure 5). However, the compounds cluster well by some of their established pharmacodynamic and pharmacokinetic differences, in that Rolipram and RS25344 are more potently emetic and somewhat more brain-penetrant than the second-generation PDE4 inhibitors Roflumilast, Piclamilast, and YM976. In addition, Rolipram and RS25344 exhibit a preference for binding to the HARBS (high-affinity Rolipram-binding state [76]) conformation of PDE4s, as well as an increased potency to inhibit the PKA-phosphorylated/activated PDE4 long forms, whereas Roflumilast, Piclamilast and YM976 do not [78,79] (Figure 5). 

### 3.4. Role of α_2_-Adrenoceptor Signaling in Body Temperature Regulation

**Figure 6:**
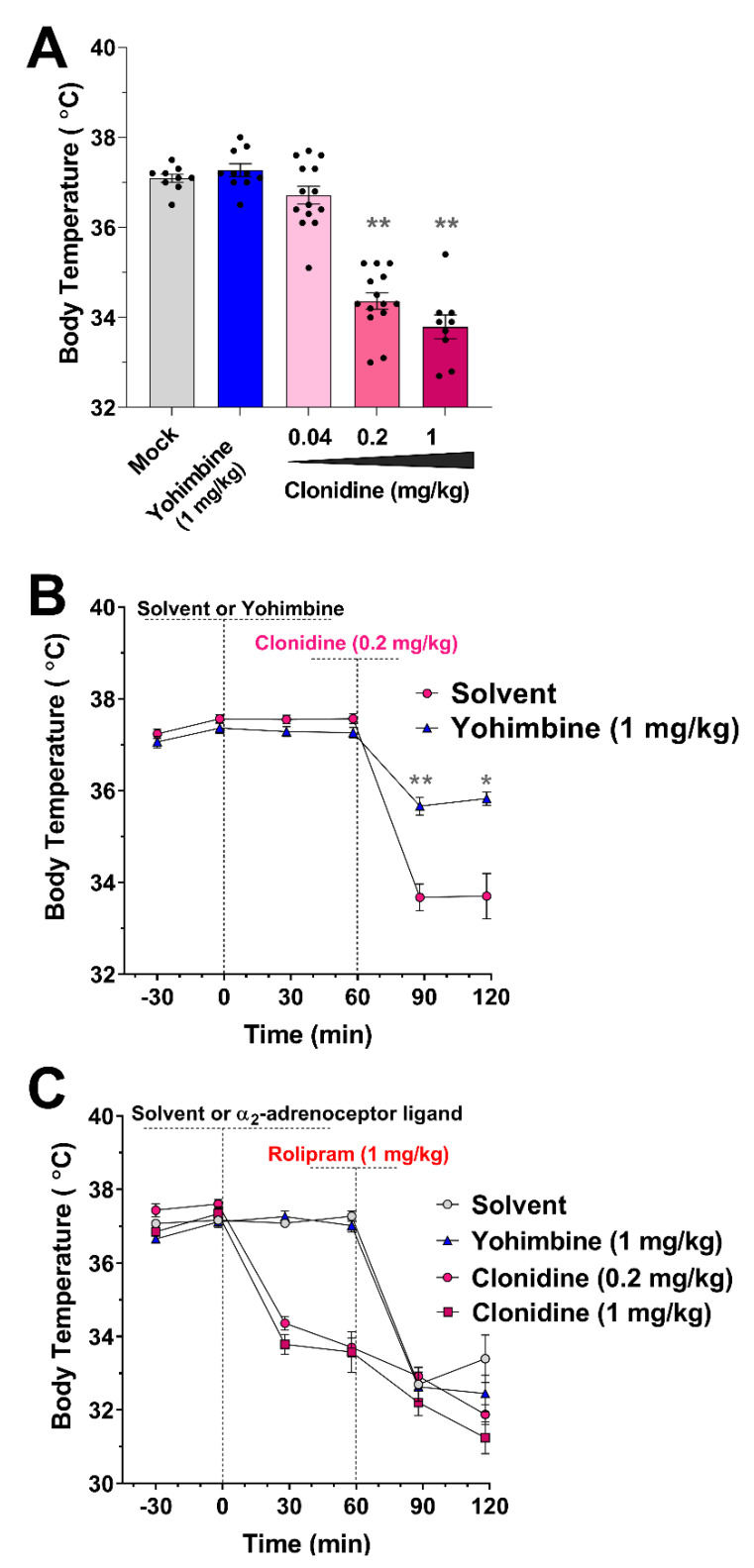
**Agonism atα_2_-adrenoceptors induces hypothermia, but neither agonism nor antagonism ofα_2_-adrenoceptors protects from PDE4 inhibitor-induced hypothermia in mice.** (**A**) The effect of treatment with the α_2_-adrenoceptor antagonist Yohimbine (1 mg/kg; i.p.) or the α_2_-adrenoceptor agonist Clonidine (0.04, 0.2 and 1 mg/kg; i.p.) on core body temperature measured 30 min after drug injection. Each dot represents a different animal. (**B**) Pre-treatment with the α_2_-adrenoceptor antagonist Yohimbine (1 mg/kg; i.p., at 0 min) protects from hypothermia induced by the α_2_-adrenoceptor agonist Clonidine (administered i.p. 60 min after pretreatment) (*n* = 8). (**C**) Neither pre-treatment with the α_2_-adrenoceptor antagonist Yohimbine (1 mg/kg; i.p.) nor the α_2_-adrenoceptor agonist Clonidine (0.2 or 1 mg/kg; i.p.) protects from or alters PDE4 inhibitor-induced hypothermia (Rolipram, 1 mg/kg, administered i.p. at 60 min after pretreatment) (*n* = 8). All data represent the mean ± SEM. Statistical significance was determined using the Kruskal–Wallis test and Dunn’s post hoc test for the bar graph in (**A**) and using two-way ANOVA with Sidak’s post hoc test for the time courses in (**B**,**C**) and is indicated as * (*p* < 0.05), or ** (*p* < 0.01).

Given that distinct PDE4 subtypes appear to mediate the effect of PAN-PDE4 inhibitors on the duration of Ketamine/Xylazine-induced anesthesia (Figure 2) or hypothermia (Figure 4 and Figure 5), we wished to assess the level of similarity or divergence of the molecular mechanisms underlying these two phenotypes. To this end, we tested whether the PDE4 inhibitors’ ability to mimic α_2_-adrenoceptor antagonism, which drives their effect on the duration of Ketamine/Xylazine-induced anesthesia, may also contribute to their induction of hypothermia. As shown in Figure 1C, treatment with the α_2_-adrenoceptor blocker Yohimbine (1 mg/kg; i.p.) mirrors the effect of PAN-PDE4 inhibitors and potently shortens the duration of Ketamine/Xylazine anesthesia. Conversely, the same dose of Yohimbine does not have any effects on baseline body temperature (Figure 6A) and thus does not replicate the effect of PAN-PDE4 inhibitors in this paradigm. Intriguingly, however, treatment with the α_2_-agonist Clonidine induced dose-dependent hypothermia in mice (Figure 6A). Clonidine-induced hypothermia was effectively alleviated by pre-treatment with the α_2_-blocker Yohimbine (Figure 6B). Conversely, neither pre-treatment with the α_2_-blocker Yohimbine nor pre-treatment with the α_2_-agonist Clonidine alleviated PAN-PDE4 inhibitor-induced hypothermia (Figure 6C), suggesting that α_2_-adrenoceptor signaling is not involved in the body temperature perturbance produced by PAN-PDE4 inhibitors. Taken together, these data suggest that distinct molecular pathways mediate the effect of PDE4 inhibitors on the two correlates of emetic potential studied here. While the effect of PAN-PDE4 inhibitors on the duration of Ketamine/Xylazine-induced anesthesia is mediated via α_2_-adrenoceptor antagonism, the effect of PDE4 inhibitors on hypothermia is independent of α_2_-adrenoceptor signaling, and it is thus understandable that distinct patterns of PDE4 subtypes are involved in these two paradigms. 

### 3.5. Treatment with the Antiemetic Metoclopramide Alleviates PAN-PDE4 Inhibitor-Induced Hypothermia in Mice

**Figure 7:**
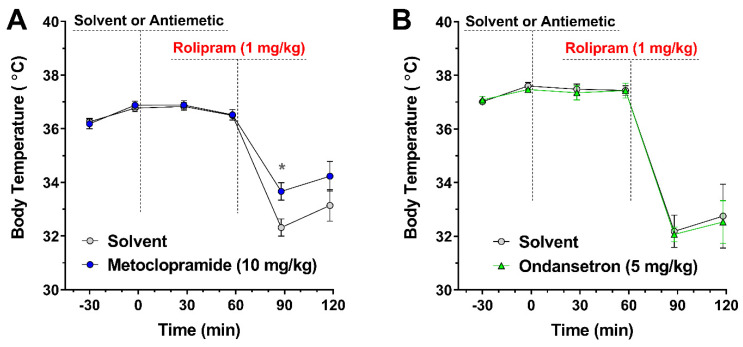
**Effect of the antiemetics Metoclopramide and Ondansetron on PDE4 inhibitor-induced hypothermia.** After measurement of baseline body temperature (at −30 and −1 min), mice were treated with the antiemetics Metoclopramide (10 mg/kg; o.g.; *n* = 12) or Ondansetron (5 mg/kg; i.p.; *n* = 6) or their respective solvent controls (Solvent), followed 60 min later by injection of the PDE4 inhibitor Rolipram (1 mg/kg; i.p.). Body temperature was measured at the indicated time points using a rectal probe thermometer. Pre-treatment with the prokinetic Metoclopramide (**A**), but not pre-treatment with Ondansetron (**B**), alleviated PDE4 inhibitor-induced hypothermia. All data represent the mean ± SEM. Statistical significance was determined using two-way ANOVA with Sidak’s post hoc test and is indicated as * (*p* < 0.05).

If hypothermia represents a close correlate of the emetic potential of PAN-PDE4 inhibition in mammals, then anti-emetic medications may also be effective in reducing PDE4 inhibitor-induced hypothermia. To test this idea, we explored the role of two anti-emetics used to treat nausea due to chemo- or radiation-therapy: the 5-hydroxytryptamine 3 (5-HT_3_)-serotonin receptor blocker Ondansetron and the prokinetic Metoclopramide, which acts via D_2_-dopamine receptor antagonism and 5-HT_4_-serotonin receptor agonism. As shown in Figure 7, treatment with Metoclopramide partially alleviated hypothermia induced by the PAN-PDE4 inhibitor Rolipram (Figure 7A), whereas Ondansetron had no effect (Figure 7B).

## 4. Discussion

### 4.1. Assessing Hypothermia as a Correlate of PDE4 Inhibitor-Induced Nausea and Emesis in Mice: Comparison of First- and Second-Generation PDE4 Inhibitors

Nausea and/or emesis induced by a variety of triggers, from motion-sickness to radiation-treatment, anesthesia and/or surgery, or treatment with distinct classes of drugs, can all produce features of impaired thermoregulation [43,44,45,46,47]. This raises the question of whether PDE4 inhibitor-induced hypothermia may also represent an accurate and thus valuable correlate of the emetic potential of this particular class of drugs. Intriguingly, the potency of PAN-PDE4 inhibitors to induce nausea and/or emesis in humans [59,76], the potency of the same drugs to induce vomiting in animals [65,80,81], as well as the potency of these drugs to induce correlates of emesis in animals, such as shortening the duration of α_2_-adrenoceptor-dependent anesthesia [52,63,64] and gastric retention [54,82], as well as hypothermia [42], all align to indicate that first-generation PDE4 inhibitors, such as Rolipram or RS25344, are significantly more emetic than second-generation PDE4 inhibitors. This pattern is confirmed in the present study. As shown in Figure 1C, the first-generation PDE4 inhibitor Rolipram is more potent than Piclamilast in the Ketamine/Xylazine anesthesia model. For hypothermia, the higher potency of first-generation PDE4 inhibitors was reported previously [42] but is also apparent in Figure 5, as doses of 0.2 mg/kg Rolipram or RS25344 (Figure 5A,B) produce a more substantial hypothermia compared to higher doses of various second-generation PDE4 inhibitors (Figure 5C–E). 

Importantly, the order of potencies by which they induce vomiting or correlates of emetic potential does not match the order of potency by which these drugs inhibit PDE4 activity in vivo or in vitro. This is illustrated by the fact that Rolipram is the least potent in inhibiting PDE4 of the drugs tested here but is one of the most emetic [53,76]. Nevertheless, there are several well-established properties that clearly distinguish first- and second-generation PDE4 inhibitors. The former, exemplified by Rolipram and RS25344, exhibit a preference for binding to HARBS (high-affinity Rolipram-binding state [76]), a particular conformation of PDE4 proteins, and both drugs also exhibit increased potency to inhibit PKA-phosphorylated/activated PDE4 compared to the non-phosphorylated enzymes [53,76,78]. Neither of these properties are shared by the second-generation PDE4 inhibitors, such as Roflumilast or Piclamilast [53,59,78,79] (Figure 5). Rolipram and RS25344 are arguably also more brain-penetrant [54,77]. Thus, it seems plausible that a preference for HARBS and/or PKA-phosphorylated/activated PDE4 and/or high brain-penetrance may increase the emetic potential of PDE4 inhibitors such that they should be avoided in drug development. As these three properties strongly pattern together, it is currently difficult to clearly identify the main driver of the high emetic potential of first-generation PDE4 inhibitors among them. Perhaps the latter is not necessary, as these three properties may define the same pool of PDE4. Given that PDE4 is expressed at high levels in the brain, that cAMP/PKA-signaling is critical for neuronal signaling and that HARBS is highly enriched in the brain [76,83,84,85,86], the high emetic potential of first-generation PDE4 inhibitors may be due to engagement of a single pool of PKA-activated, neuronal PDE4 that preferentially exists in HARBS conformation. Taken together, PDE4 inhibitor-induced hypothermia in mice replicates differences in the emetic potential of first- and second-generation PDE4 inhibitors that are well-established in humans and animals, suggesting that hypothermia in mice may represent a useful correlate of the emetic potential of PDE4 inhibitors. This conclusion is further supported by the finding that treatment with a clinically used anti-emetic, Metoclopramide (Figure 7A), alleviates PDE4 inhibitor-induced hypothermia. That both anti-emetics tested here did not alleviate PDE4 inhibitor-induced hypothermia (e.g., Ondansetron; Figure 7B) does not refute this argument, as it is well-known that most anti-emetics do not exhibit a broad-spectrum clinical efficacy. Instead, the efficacy of a particular class of anti-emetics is generally closely tied to the particular cause and hence the molecular mechanism that induces nausea/emesis. Prior reports have shown that this concept extends to the efficacy of anti-emetics to alleviate hypothermia induced by distinct triggers, such as provocative motion, radiation, or drugs [37,48,49,87]. 

### 4.2. Role of Individual PDE4 Subtypes in Mediating the Side Effects of PAN-PDE4 Inhibitors: Comparison of Distinct Correlates of Emetic Potential in Mice

Given that development of subtype-selective PDE4 inhibitors is a promising approach to mitigate the adverse effects of PAN-PDE4 inhibitors, while retaining their therapeutic benefits, we wished to employ hypothermia in mice as a correlate to identify the specific PDE4 subtype(s) that predominantly mediate(s) the emetic potential of PDE4 inhibitors. To this end, we assessed body temperatures in mice deficient in each of the four PDE4 subtypes, PDE4A to D (Figure 4). These experiments revealed that selective inactivation of each PDE4 subtype *per se* does not produce hypothermia, which instead likely results from the concurrent inhibition of multiple (at least two) PDE4 subtypes. Alternatively, one could also postulate that a critical role of a particular PDE4 subtype on body temperature may have been obscured in the response of the respective PDE4 knockout mouse by compensatory changes in the expression or activity of the three other PDE4 isoforms. We consider this unlikely, however, given the large body of data suggesting that PDEs are not functionally interchangeable [3,88,89] and because such compensatory changes in PDE4 expression were not observed in various primary cells or tissues of PDE4-KO mice reported previously [19,20,22,23,52,61,90,91,92]. 

The finding that ablation of any individual PDE4 subtype does not replicate PAN-PDE4 inhibitor-induced hypothermia mirrors the effect of subtype-selective PDE4 ablation on gastric retention reported previously [82]. Moreover, pre-treatment with the pro-kinetic Metoclopramide has been shown to alleviate acute gastric retention induced by PAN-PDE4 inhibition [82] and also alleviates PAN-PDE4 inhibitor-induced hypothermia (Figure 7). Given prior reports that blockade of D_2/3_-dopamine receptors alleviates PDE4 inhibitor-induced hypothermia [42], this may suggest that Metoclopramide, which has some affinity for multiple receptor classes, including D_2_-dopamine, muscarinic and serotonergic receptors [93,94,95], alleviates both gastric retention and hypothermia *via* its blockade of D_2/3_-dopamine receptors. 

In contrast to the effect of subtype-selective PDE4 ablation on body temperature and gastric motility, the effect of PAN-PDE4 inhibition on the duration of α_2_-adrenoceptor-dependent anesthesia [63,64,65] is exclusively mediated and replicated by inactivation of PDE4D [52] (Figure 2). This difference in the role of the PDE4D subtype is paralleled by distinct molecular mechanisms involved in these correlates of emetic potential. The Ketamine/Xylazine model tests whether PDE4/PDE4D inactivation can act via physiological α_2_-adrenoceptor antagonism to shorten/counteract α_2_-adrenoceptor-dependent anesthesia (see Figure 1C). Conversely, as shown in Figure 6, PDE4 inhibitor-induced hypothermia is not mediated by or dependent upon α_2_-adrenoceptor antagonism. Intriguingly, both hypothermia as well as gastric retention [82] induced by PDE4 inhibitors are unaffected by α_2_-adrenoceptor antagonism (Figure 6A), whereas α_2_-adrenoceptor agonists, such as Clonidine, induce hypothermia (Figure 6A) as well as gastric retention [82]. Thus, gastric retention and hypothermia share multiple molecular mechanisms, including the absence of a predominant role of PDE4D and the effect of D_2/3_-dopamine receptor antagonists, as well as the effects of α_2_-adrenoceptor agonists and antagonists in these paradigms.

The role of PDE4D in curtailing α_2_-adrenoceptor-dependent anesthesia may lead to the assumption that inhibition of PDE4D is irrevocably tied to adverse effects and that any therapeutic benefits that may be derived from targeting PDE4D must be forfeited. In contrast, the observation that the concurrent inhibition of multiple PDE4 subtypes is required to induce hypothermia (Figure 4) or gastric retention suggests that the selective inhibition of any individual PDE4 subtype, including the selective inhibition of PDE4D, may be free of nausea and emesis. This may hold true even if PDE4D should be one of the PDE4 subtypes that must be inhibited simultaneously to produce these adverse effects, and thus suggests that PDE4D may be targeted for therapeutic benefits. This idea is validated to some extent by the reduced emetogenic potential of allosteric PDE4D-selective inhibitors in animal models reported previously [96,97].

Considering the distinct effect of PDE4D ablation on Ketamine/Xylazine-induced anesthesia *versus* hypothermia, it is tempting to speculate which of the two is a more accurate correlate of emetic potential. However, as a short historic perspective on the emetic effects of other drug classes (e.g., chemotherapeutics [37]) would quickly reveal, such speculations are likely futile and error-prone at present. Only after candidate PDE4 inhibitors (whether they are designed to be subtype/PDE4D-selective, or perhaps conformation-selective) demonstrate the absence of adverse effects in humans may these compounds also serve to identify the more appropriate animal model that reflects the emetic potential of PDE4 inhibition, which may subsequently serve as a standard in drug development. Moreover, just as individual patients taking PDE4 inhibitors may experience variable levels of nausea and emesis, it is also possible that distinct molecular mechanisms contribute to nausea and emesis in individual patients experiencing these adverse effects, which opens the possibility that different animal models capture the emetic potential of PDE4 inhibitors reflective of distinct patient populations. 

### 4.3. Variable Responses of PDE4D-KO Mice to Hypothermia Induced by First- and Second-Generation PAN-PDE4 Inhibitors

The observation that two first-generation PDE4 inhibitors, Rolipram and RS25344 (Figure 5A,B), produce less severe hypothermia in PDE4D-KO mice compared to wildtype controls is noteworthy, given that this involves PDE4D somehow in the hypothermia paradigm. There are various possible explanations for this observation, all of which remain to be tested experimentally, however. For example, given the necessity of maintaining normal body temperature for the survival of the organism, one may speculate that the long-term deletion of PDE4D in the KO animals, even if acute PDE4D inactivation were to affect body temperature, would trigger an adaptation and compensatory mechanisms to restore body temperature back to 37 °C. Even if so, PDE4D must not play a predominant role, however, given that all PDE4 inhibitors, including the first-generation PDE4 inhibitors Rolipram and RS25344, produce significant hypothermia in PDE4D-KO mice. This clearly shows that these inhibitors act in PDE4D-KO mice *via* inhibition of PDE4s other than PDE4D to induced hypothermia. But why are there differences between first-generation PDE4 inhibitors, which distinguish between WT and PDE4D-KO mice, and second-generation PDE4 inhibitors, which do not? These differences suggest that while PDE4D is mechanistically linked to the highly emetic effects exhibited by Rolipram and RS25344, there is no such mechanistic link associating PDE4D with the emetic potential that remains in current second-generation PDE4 inhibitors. In other words, first- and second-generation PDE4 inhibitors induce emesis *via* inhibition of distinct, though likely overlapping, pools of PDE4 in the body. By avoiding a particular pool of PDE4, which is likely predominated by PDE4D (e.g., a pool of PDE4D in HARBS conformation in the central nervous system), second-generation PDE4 inhibitors present with an improved safety profile. However, PDE4D does not seem to play a predominant role in mediating the remaining adverse effects of second-generation PDE4 inhibitors. Thus, for further improvement in the safety profile of this class of drugs (e.g., development of third-generation PDE4 inhibitors) there is little concrete evidence to suggest that their action on PDE4D must be avoided. 

## 5. Conclusions

Given the growing body of evidence that links nausea and emesis to disturbances in thermoregulation in mammals, we here explored PDE4 inhibitor-induced hypothermia as a novel correlate of nausea in mice. Using knockout mice for each of the four PDE4 subtypes, we show that selective inactivation of individual PDE4 subtypes does not produce hypothermia, which must instead require the concurrent inactivation of multiple (at least two) PDE4 subtypes. This finding contrasts with prior reports that proposed PDE4D as the subtype mediating these adverse effects of PAN-PDE4 inhibitors and suggests that inhibitors that selectively target any individual PDE4 subtype, including those targeting PDE4D, may be free of nausea and emesis.

Intriguingly, the potency of distinct PDE4 inhibitors to induce hypothermia correlates well with the reported potency of these drugs to induce nausea and/or emesis in humans, as well as their potency to engage correlates of emetic potential in animals. In addition, hypothermia mirrors some of the facets of PDE4 inhibitor-induced gastric retention reported previously [82], in that both require the inactivation of multiple PDE4 subtypes and both are alleviated by D_2/3_-dopamine receptor blockers. Finally, treatment with a clinically used anti-emetic, Metoclopramide, alleviates PDE4 inhibitor-induced hypothermia in mice, providing a mechanistic link between the two physiological paradigms. Together, these observations suggest that hypothermia may represent a useful correlate for PDE4 inhibitor-induced nausea and thus represents a rapid and cost-effective experimental approach to evaluate novel lead compounds or to explore novel mechanistic insights to facilitate drug development. 

## Data Availability

Not applicable.

## Abbreviations

cAMP, 3’,5’-cyclic adenosine monophosphate; DMSO, dimethyl sulfoxide; HARBS, high-affinity Rolipram-binding state; i.p., intraperitoneal; KO, knockout; o.g., oral gavage; PBS, phosphate-buffered saline; PDE, cyclic nucleotide phosphodiesterase; PDE4, cAMP-phosphodiesterase 4; PKA, protein kinase A; PONV, post-operative nausea and vomiting; WT, wildtype.
